# “If we miss this chance, it’s futile later on” – late antenatal booking and its determinants in Bhutan: a mixed-methods study

**DOI:** 10.1186/s12884-019-2308-5

**Published:** 2019-05-07

**Authors:** Thinley Dorji, Mrinalini Das, Rafael Van den Bergh, Myo Minn Oo, Sonam Gyamtsho, Karma Tenzin, Tashi Tshomo, Sonam Ugen

**Affiliations:** 1 Jigme Dorji Wangchuck National Referral Hospital, Thimphu, Bhutan; 2 Kidu Mobile Medical Unit, His Majesty’s People’s Project, Thimphu, Bhutan; 3 Médecins Sans Frontières, Operational Centre Brussels, New Delhi, India; 4 Médecins Sans Frontières, Operational Centre Brussels, Operational Research Unit (LuxOR), Luxembourg City, Luxembourg; 5 The International Union of Tuberculosis and Lung Diseases, Mandalay, Myanmar; 6 Khesar Gyalpo University of Medical Sciences of Bhutan, Thimphu, Bhutan; 7 Reproductive Health Programme, Ministry of Health, Thimphu, Bhutan

**Keywords:** Maternal and child health, Maternal mortality, Positive pregnancy experience, Abortion, Health seeking barrier, Asia

## Abstract

**Background:**

To achieve the Sustainable Development Goal related to maternal and neonatal outcomes, the World Health Organization advocates for a first antenatal care (ANC) contact before 12 weeks of gestation. In order to guide interventions to achieve early ANC in the lower middle-income setting of Bhutan, we conducted an assessment of the magnitude and determinants of late ANC in this context.

**Methods:**

This was a mixed-methods study with quantitative (cross-sectional study) and qualitative (in-depth interviews with pregnant women and ANC providers) component in a concurrent triangulation design. The quantitative component retrospectively analysed the socio-demographic and clinical characteristics, and the gestational age at booking of women who were provided care for delivery or miscarriages at the three tertiary hospitals in Bhutan from May–August 2018. The qualitative component involved thematic analysis of in-depth interviews with ten women attending ANC visits and four healthcare workers involved in ANC provision.

**Results:**

Among 868 women studied, 67% (*n* = 584) had a late booking (after 12 weeks), and 1% (*n* = 13) had no booking. Women with only primary education and those residing in rural areas were more likely to have a late first ANC booking. While many women achieved the recommended eight ANC visits, this did not necessarily reflect early booking. Late booking was common among multigravida women. The interviews illustrated a general understanding and recognition of the importance of early ANC. Support from peers, family and co-workers, and male participation in accessing ANC were seen as enablers. The outreach clinics (ORCs) at the primary healthcare level were an important means of reaching the ANC services to women in rural areas where geographical accessibility was a barrier. Specific barriers to early ANC were gender insensitivity in providing care through male health workers, cost/time in ANC visits, and the inability to produce the documents of the father for booking ANC.

**Conclusion:**

Late ANC booking was common in Bhutan, and appeared to be associated with educational, geographic, socio-cultural and administrative characteristics. A comprehensive information package on ANC needs to be developed for pregnant mothers, and the quality of ANC coverage needs to be measured in terms of early ANC booking.

**Electronic supplementary material:**

The online version of this article (10.1186/s12884-019-2308-5) contains supplementary material, which is available to authorized users.

## Background

Maternal and child health (MCH) are central to achieving the Sustainable Development Goal 3, related to maternal and neonatal outcomes of pregnancy [1]. As part of the global strategy to reduce the risk of stillbirth and maternal complications, the World Health Organization (WHO) aims to improve the quality of antenatal care by increasing the number of recommended visits (from four to eight), and advocating for a first ANC contact (also known as the booking visit) before 12 weeks of gestation. Early provision of ANC has been found crucial for ensuring optimal care and good maternal and foetal health [2]. However, many countries, and low- and middle-income countries (LMIC) in particular, face challenges in implementing the recommended number and timing of such visits [3–6].

Bhutan is such a country stepping up the quality of its ANC package. Bhutan has seen a steady decrease in maternal mortality, from 255 deaths per 100,000 live births in 2000 to 86 per 100,000 live births in 2012 [7]. The infant mortality rate has also reduced from 61 per 1000 live births in 2000 to 30 per 1000 live births in 2012 [7]. The neonatal mortality rate was 21 per 1000 live births in 2012 [7]. The rate of stillbirth in 2015 among institutional deliveries, which covered 86% of all births in the country, was 10 per 1000 live births, much lower than the global estimate of 18.4 per 1000 live births [8, 9]. Effective ANC services are required to sustain these positive trends. ANC reduces maternal and perinatal morbidity and mortality both directly, through detection and treatment of pregnancy-related complications, and indirectly, through the identification of women at increased risk of developing complications during labour and delivery, thus ensuring referral to an appropriate level of care [10, 11]. The Ministry of Health, Royal Government of Bhutan guideline 2010 and the WHO ANC Model 2016 recommend a minimum of eight ANC contacts, with the booking visit preferably scheduled within the first trimester [10].

An early booking visit for ANC has thus been prioritized by Bhutan. In the national MCH Programme, the initial ANC visit involves health education on pregnancy and exclusive breast feeding practice; provider-initiated screening for syphilis, hepatitis B and HIV; provision of micronutrient supplements; advice on the birth preparedness plan; and encouraging women to deliver in health centres [12]. ANC is given free of charge by the Royal Government. Reports suggest that the majority of women have their initial booking visit only in the second or the third trimester, which may place the pregnant woman and her child at risk of complications before and during birth [13].

A number of studies in LMIC have explored the underlying reasons of late ANC booking visits, the definition of which, prior to the WHO 2016 guideline, varied from later than 14 to 20 weeks of gestation [3–5, 10, 14]. The level of education and income, having an unintended pregnancy, living in rural areas, age of the mother, parity, and the perception that healthcare visits are not required if there are no acute problems during pregnancy were documented factors associated with late ANC booking [3–6]. While such studies are generalizable to an extent, they may not capture the socio-cultural specificities of a context such as Bhutan. In order to fully explore the magnitude, as well as the barriers and enablers, of early ANC booking, we conducted a mixed-methods study on the characteristics of early versus late ANC booking in Bhutan, and the healthcare worker and beneficiary perspectives on early ANC booking in this context.

## Methods

### Study design

This was a mixed-methods study with a quantitative (cross-sectional study) and qualitative (in-depth interviews with pregnant women and ANC providers) component in a concurrent triangulation design.

### Setting

#### General setting

Bhutan is a small country situated in the eastern Himalayas with a population of 0.7 million [15]. The crude birth rate is 16 per thousand population and the total fertility rate is 1.7 [15]. Healthcare is provided free of cost at all levels, guided by the National Health Policy 2011 within the broader framework of overall national development and pursuit of happiness [16, 17]. Healthcare is provided through a three-tiered system: basic health units and outreach clinics at the primary level, district and general hospitals at the secondary level, and referral hospitals with specialist obstetrics services at the tertiary level [13]. The three referral centres are located in geographically strategic locations in the west, east and central regions.

#### Maternal and Child Health Programme

MCH services are provided through the healthcare system after confirmation of pregnancy by urine beta-hCG test or ultrasound scan. The package of antenatal care is the same at all levels of health centres. The care during the antenatal period and the so-called ‘Thousand Golden Days’ is monitored using the MCH Handbook [12]. An ANC coverage of at least one visit was recorded in 97.9% of 2144 women surveyed in the National Health Survey of 2012, but the coverage of at least eight visits was only 26.1% [7].

Briefly, after confirmation of pregnancy, women visit the MCH unit at the nearest health centre, where the pregnancy is registered by Health Assistants and/or Nurses. A personal MCH Handbook is issued, and baseline socio-demographic and clinical data are recorded in this Handbook; a copy of the information is also maintained in the MCH Register, which remains at the facility. During each antenatal visit, the health status of the mother and the foetus is assessed and recorded in both the personal MCH Handbook and in the MCH Register and the online MCH Tracking System.

The details of the birth are recorded in the MCH Handbook and the Birth Register in the obstetrics ward. The Birth Register is completed at the time of delivery with essential information from the MCH Handbook, including the ANC booking date.

### Study site

This study was conducted in the Maternity Wards of the three tertiary care hospitals in Bhutan: the Jigme Dorji Wangchuck National Referral Hospital, Thimphu; the Central Regional Referral Hospital, Gelegphu and the Eastern Regional Referral Hospital, Monggar. Interviews were conducted at the Jigme Dorji Wangchuck National Referral Hospital and Damphu Hospital in Tsirang.

### Study population

Quantitative: All pregnant women who delivered or were provided care for miscarriage or other complications leading to a pregnancy outcome in the second or third trimester, from 15 May to 14 August 2018 were included, and a formal sample size was not calculated. These centres cover approximately 58% of all deliveries in Bhutan [13, 18].

Qualitative: Purposive sampling was conducted among pregnant women (receiving antenatal care between 15 May and 14 August 2018) and healthcare professionals. For pregnant women, maximal variation was sought for early versus late booking and for gravidity. The mean age of the included mothers was 28 years; five were primigravida, four were second gravida and one was an elderly mother in fifth gravida. Healthcare workers (Health Assistants and Nurses) with a minimum of 5 years of experience working and having served in at least one rural health centre were selected. The mean duration of work experience of the health workers was 15 years and all had served in at least two primary health centres (Basic Health Unit Grade II). One pregnant mother and one healthcare worker who refused to provide consent for the interview were excluded from this study component. The participants did not know the interviewer; they met the interviewer face to face at the time of interview.

### Data variables, sources and data collection

For the quantitative component, data were sourced from the Birth Register and admission register of the Maternity Wards. Data variables included the date and socio-demographic, clinical and pregnancy characteristics at the time of booking; date and gestational age at pregnancy outcome (delivery or miscarriage); and the number of ANC visits in the current pregnancy. These were extracted into a structured pro forma (Additional file 1).

For the qualitative component, in-depth interviews were held with ten pregnant women and four healthcare providers about their experiences with ANC and the possible barriers and enablers for early booking visits during pregnancy. Prior to the interview, the participants were given written and verbal information on the study purpose and process. Informed consent was then taken for the interview and for audio-recording.

The pregnant women who presented at the Jigme Dorji Wangchuck National Referral Hospital, Thimphu were selected purposively by the MCH doctor as described above, and the selected participants were directed to the principal investigator. The interviews were conducted in one of the consultation chambers that was not in use. For two mothers, interviews were conducted in the presence of companions (husband in one case, sister in the other case) as these mothers reported that they felt comfortable to have a companion inside the interview room.

Two ANC providers from a tertiary hospital (one Health Assistant and one Nurse) and two from primary a health centre (one Health Assistant and one Nurse) were also interviewed after taking informed consent. The interviews were carried out on the premises of the MCH Unit in a room where privacy was ensured; only the participants and the researcher were present.

The interviews were conducted based on a guide (Additional file 2) that was pilot tested with three pregnant women at the Jigme Dorji Wangchuck National Referral Hospital, not included in the current study. The pilot test sessions were attended by KT and SU, who are experienced in qualitative research. The flexibility in the conduct of the interviews allowed for open discussion and re-ordering of the questions from the interview guide according to the flow of conversation with the participant. No repeat interviews were conducted. Data collection was continued until saturation was achieved, i.e. when the data obtained began to present redundancy or repetition. The interview session with each participant lasted between 15 to 20 minutes. All interviews were conducted by the principal investigator (male, general doctor with Bachelor of Medicine and Bachelor of Surgery (MBBS) degree working at the Jigme Dorji Wangchuck National Referral Hospital, Thimphu), and in the local language (Dzongkha and Sharchokpa in which the principal interviewer was fluent). The questions were easy to understand and presented in everyday language. Field notes were taken during the interview. The interviews were audio-recorded and transcribed in English by the principal investigator on the same day of the interview and information was corroborated with the field notes. The audio files were deleted after transcription.

### Data analysis and statistics

#### Quantitative

Data collected in the structured form were double entered, validated and analyzed (unadjusted analysis) using EpiData (version 3.1 for entry and version 2.2.2.183 for analysis, EpiData Association, Odense, Denmark). The adjusted analysis was done using STATA (version 12.1, copyright 1985–2011 StataCorp LP USA, serial number: 30120504773). Socio-demographic and clinical characteristics were summarized using frequencies and proportions for categorical variables. Gestational age in weeks at ANC booking was calculated from the gestational age at delivery and the duration between date of ANC booking and date of delivery (in weeks). Those who had ANC booking after first trimester or did not have ANC booking at all were regarded as late ANC booking. Associations between socio-demographic and clinical characteristics and late ANC booking were assessed using unadjusted prevalence ratios (PR) with their associated 95% confidence intervals (95%CI). Those risk factors with a *p*-value< 0.1 were included in the adjusted analysis using a log-binomial regression model. An adjusted p-value < 0.05 was considered significant.

#### Qualitative

Thematic analysis was carried out manually and themes and subthemes related to the perception of ANC services and booking visits during ANC were developed by two independent researchers (TD and RVdB) [19, 20]. No pre-defined themes were used. Transcripts and themes were reviewed by a third person (MD) to reduce subjective bias and enhance interpretive credibility. Disagreements in the thematic analyses were resolved through discussion. To anonymise the quotations that were used to illustrate the themes, mothers are identified with their gravida and age range, and healthcare workers as HCW.

### Ethics considerations

Ethics approval was obtained from the Ethics Advisory Group of The Union, Paris, France, and Research Ethics Board of Health, Ministry of Health, Thimphu, Bhutan. Permission and support for the study were sought from the hospital administrators before initiation. As the study involved review of existing MCH records, a waiver for informed consent was sought and approved by the ethics committees. For the in-depth interviews, written informed consent was taken from the study participants as per the consent process submitted to the ethics committees. For the quantitative component, personal identifiers were removed after validation and all analyses were performed on the anonymised dataset. Only anonymised data are presented in this paper.

## Results

**Fig. 1:**
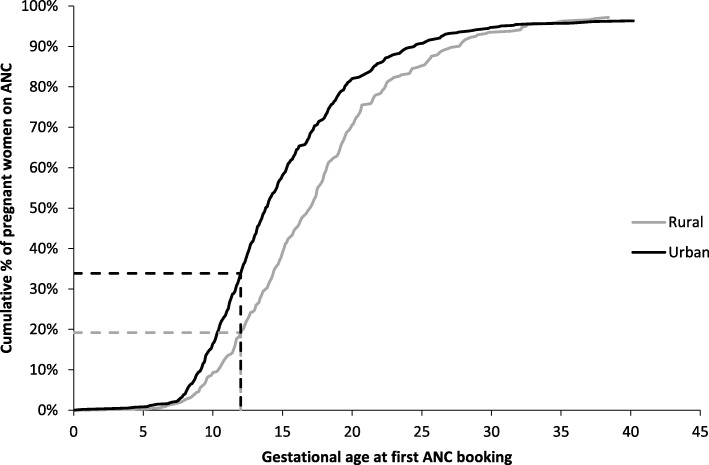
Antenatal care coverage by gestational age at first booking among women who delivered or presented for hospital care leading to a pregnancy outcome in three tertiary hospitals, Bhutan, May–August 2018

From 1661 women who received care for a delivery or miscarriage at the three tertiary hospitals during the study period, 868 had valid information on the timing of the first ANC booking. The cumulative proportion of women having their first ANC booking in the function of their gestational age, stratified by their residence type (rural versus urban) is provided in Fig. 1: while a high ANC coverage (> 95%) of at least one visit was reached in both rural and urban contexts, rural contexts were observed to have a later first ANC booking. Among the 868 records, the proportion of women who had a late booking (after 12 weeks) was 67% (*n* = 584), and of women with no booking was 1% (*n* = 13). The socio-demographic, clinical, and pregnancy characteristics are given in Tables 1 and 2 respectively.

**Table 1:** Socio-demographic and clinical characteristics of women who delivered or presented for hospital care leading to a pregnancy outcome in three tertiary hospitals, Bhutan, May–August 2018

Categories	n	(%)
Total	868	(100)
Age		
16–17 years	7	(< 1)
18–24 years	178	(21)
25–34 years	561	(65)
35–44 years	118	(14)
≥ 45 years	2	(< 1)
Not recorded	2	(< 1)
Level of education of mother		
None	165	(19)
Primary education	81	(9)
Secondary education	121	(14)
Graduate education	369	(43)
Non-formal education	126	(15)
Others	2	(< 1)
Not recorded	4	(< 1)
Residence		
Urban	545	(63)
Rural	323	(37)
Past medical history of mother		
Hypertension	32	(4)
Known substance or alcohol abuse	3	(< 1)
Diabetes	2	(< 1)
Tuberculosis	2	(< 1)
Past obstetric history		
Mother is Rh-negative	7	(1)
Bad obstetric history	1	(< 1)
Low birth weight	9	(1)
High birth weight	1	(< 1)
Hospital admission in last pregnancy	2	(< 1)
Previous pelvic surgery	122	(14)
HIV infection		
Negative	859	(99)
Positive	1	(< 1)
Not recorded	8	(< 1)
Hepatitis B infection		
Negative	849	(98)
Positive	9	(1)
Not recorded	10	(1)
ANC booking category		
Early booking	271	(31)
Late booking	584	(67)
No booking	13	(1)

Bad obstetric history = History of three or more consecutive spontaneous abortions; Low birth weight = Baby weighing less than 2500 g; High birth weight = Baby weighing more than 4500 g; Reasons for hospital admissions in last pregnancy = admission for hypertension, preeclampsia, eclampsia; Previous pelvic surgery = Surgery on reproductive tract such as caesarean section, cervical cerclage, cone biopsy, myomectomy, ectopic pregnancy

*ANC* antenatal care, *BMI* body mass index

**Table 2:** Pregnancy characteristics of women who delivered or presented for hospital care leading to a pregnancy outcome in three tertiary hospitals, Bhutan, May–August 2018

Categories	n	(%)
Gravidity		
Primigravida	323	(37)
Multigravida	545	(63)
Parity		
Nullipara	343	(40)
Primipara (P1)	302	(35)
Multipara (P2-P4)	210	(24)
Grand-multipara (≥P5)	13	(1)
Number of living children (*n* = 545)		
0	46	(8)
1	305	(56)
2–3	178	(33)
≥ 4	16	(3)
History of abortion (*n* = 545)		
0	478	(87)
1	59	(11)
2–4	8	(1)
History of stillbirth (*n* = 545)		
0	538	(98)
1	6	(1)
2	1	(< 1)
Number of dead children (*n* = 545)		
0	478	(87)
1	53	(10)
2	11	(2)
3–4	3	(1)
Number of antenatal visits at delivery/pregnancy outcome		
0 (no booking)	13	(< 1)
1	3	(< 1)
2	8	(1)
3	21	(2)
4	47	(5)
5	62	(7)
6	126	(15)
7	133	(15)
≥ 8	440	(51)
Not recorded	15	(2)

Women with only primary education (adjusted PR 1.2, 95%CI 1.0–1.4, *p* = 0.034) and those residing in rural areas (adjusted PR 1.2, 95%CI 1.1–1.4, *p* < 0.001) were more likely to have a late first ANC booking (Table 3).

**Table 3:** ANC booking date and socio-demographic and clinical factors of women who deliverd or presented for hospital care leading to a pregnancy outcome in three tertiary hospitals, Bhutan, May–August 2018 (*N* = 868)

Categories	Early ANC Booking (n, %)	Late ANC booking (n, %)	Unadjusted analyses		Adjusted analyses	
			PR (95% CI)	p-value	aPR (95% CI)	*p*-value
Age (*n* = 866)						
16–17 years	2 (29)	5 (71)	1.0 (0.6–1.6)	0.944		
18–24 years	53 (30)	125 (70)	Ref	–		
25–34 years	183 (33)	378 (77)	1.0 (0.9–1.1)	0.468		
≥ 35 years	32 (27)	88 (73)	1.0 (0.9–1.2)	0.556		
Gravidity						
Primigravida	118 (37)	205 (63)	Ref	–		
Multigravida	153 (28)	392 (72)	1.1 (1.0–1.2)	0.012	1.0 (0.7–1.3)	0.780
Parity						
Nullipara	126 (37)	217 (63)	Ref	–		
Primipara (P1)	97 (32)	205 (68)	1.1 (1.0–1.2)	0.217	1.1 (0.8–1.5)	0.544
Multipara (P2-P4)	46 (22)	164 (78)	1.2 (1.1–1.4)	< 0.001	1.2 (0.9–1.7)	0.251
Grand-multipara (≥P5)	2 (15)	11 (85)	1.3 (1.0–1.7)	0.020	1.1 (0.7–1.8)	0.501
Level of education of mother (n = 866)						
None	41 (25)	124 (75)	1.3 (1.1–1.5)	0.005	1.1 (0.9–1.3)	0.189
Non-formal education	24 (30)	57 (70)	1.2 (1.0–1.5)	0.082	1.0 (0.8–1.2)	0.958
Primary education	26 (21)	95 (79)	1.3 (1.1–1.6)	0.001	**1.2 (1.0–1.4)**	**0.034**
Secondary education	124 (34)	245 (66)	1.1 (1.0–1.3)	0.141	1.1 (0.9–1.3)	0.252
Graduate education	52 (41)	74 (59)	Ref	–		
Others	2 (100)	0 (0)	–	–	–	0.982
Residence						
Urban	204 (37)	341 (63)	Ref	–		
Rural	67 (21)	256 (79)	1.2 (1.1–1.4)	< 0.001	**1.2 (1.1–1.4)**	**< 0.001**
Pre-existing hypertension in mother						
Yes	15 (47)	17 (53)	0.8 (0.6–1.1)	0.111	–	–
No	256 (31)	580 (69)	Ref	–		
Mother Rh negative (*n* = 862)						
Yes	2 (29)	5 (71)	1.0 (0.7–1.7)	0.875	–	–
No	267 (31)	588 (69)	Ref	–		
Past low birth weight baby						
Yes	1 (11)	8 (89)	1.3 (1.0–1.6)	0.031	1.1 (0.8–1.5)	0.647
No	270 (31)	589 (69)	Ref	–		
Hepatitis B infection (*n* = 858)						
Negative	264 (31)	585 (69)	Ref	0.471		
Positive	4 (44)	5 (56)	0.8 (0.4–1.4)	–	–	–

*PR* prevalence ratio, *aPR* adjusted prevalence ratio, Low birth weight = Baby weighing less than 2500 g; Non-formal education = certificate level course

A series of interviews were conducted with healthcare workers and pregnant women to further explore these quantitative observations. Themes around the importance of early ANC booking, making decisions for the first ANC contact, and the role of healthcare workers in influencing the first ANC contact are presented. Availability of peer and community support was identified as an enabler for early ANC booking, while geographic inaccessibility, gender sensitivity, cost and time involved in making ANC visits, as well as the need for documents for registration for ANC were identified as barriers.

### Early ANC: Recognizing the relevance

The interviews with expecting mothers and those with healthcare workers illustrated a general understanding and recognition of the importance of (early) ANC visits especially in centres that are located far away from well-equipped health centres. In the words of a healthcare worker:

*“This is the crucial time to give care. If we miss this chance, it’s futile later on. It is equally important for mothers both in the urban or rural areas, educated or uneducated” – HCW, age range 41–50 years.*

Expecting mothers expressed similar thoughts on the importance of ANC visits:

*“Early first visit is very important. That is the time you can take medicines and doctors and nurses have time to take good care of you and your baby.” – second gravida, age range 21–30 years.*

*“I know that it is important for both the mother and the baby. They do check up here and give us medicines that will help in the growth of my baby.” – primigravida, age range 21–30 years.*

*“Though I have to come again and again, I know it is for the good of self and it is important for my baby.” – fifth gravida, age range 31–40 years.*

Healthcare workers reported the relevance of ANC visits especially in centres that are located far away from well-equipped health centres:

*“Some of these women who were airlifted to the National Referral Hospital were those who did not have any ANC visits.” – HCW, age range 31–40 years.*

### Awareness and decision making

Mothers, in particular, commented on the process through which they received information, and the decisional process on making the first ANC contact. In the majority, the primary decision maker for making the booking ANC visit was the mother herself while family members also played an important role.

*“My mother and my sister told me that if I were pregnant, I should go to the hospital.” – primigravida, age range 21–30 years.*

In rural areas, ORCs at the primary level were an effective means to provide ANC education and services to women:

“*We conducted out-reach clinics from time to time. For a pastoral community in northern Bhutan, ORCs were conducted in the pastures to provide ANC services.” – HCW who served in Lunana, one of the remotest villages, eight days walk from the nearest road point.*

*“Now people know the services provided through ORC and they come forward [to utilize the services]. We take this opportunity to give health information and give antenatal services.” – HCW, age range 31–40 years.*

Whereas for mothers in urban areas, experienced family members and friends, the internet, and healthcare workers all played crucial roles in providing information and guidance.

*“I go online and look for information, and also ask my friends who have become mothers.” – primigravida, age range 21–30 years.*

*“My friends have told me that these exercises [maternal exercise programme] are very helpful and it has helped them.” – second gravida, age range 21–30 years.*

*“I don’t remember learning about it [need for antenatal visits] in school” – primigravida, age range 21–30 years.*

However, the content of health information from friends was sometimes incorrect:

*“I asked my friends when I should visit the hospital [for booking]. They told me that I can go to the hospital at any month, be it five months or even seven months.” – second gravida, age range 21–30 years.*

### Role of healthcare facilities

In many rural places, facilities to test for pregnancy are only available in health centres. The linkage of the ANC visits with pregnancy testing was emphasised:

*“Even in Trashigang town [two days drive from the capital city], pregnancy test kits are not always available from the private pharmacies.” – primigravida, age range 21–30 years.*

*“After I missed my period, I didn’t know I was pregnant. So I came here and asked the nurses whether I was pregnant or not.” – primigravida, age range 21–30 years.*

Even in urban areas, diagnosis of pregnancy was confirmed by general doctors at the hospitals [no private clinics in Bhutan] and then the mothers were sent for booking at the MCH units:

*“I visited the General Out-Patient Department. The doctor there prescribed the urine test… and a [health worker] directed me here to the Community Health Department for booking my pregnancy.” – primigravida, age range 31–40 years.*

### Enablers and barriers to (early) ANC booking

Both healthcare workers and expecting mothers contributed a series of perceived enablers and barriers for (early) ANC booking.

### Enablers of early ANC booking

- Peer & community support

Peer and family support influenced both the first and subsequent antenatal visits.

*“My relatives and friends who have delivered before me told me to make the antenatal book.” – second gravida, age range 21–30 years.*

In addition, women invited male participation in availing the ANC services and availability of such have been reported as an enabler.

*“Husbands also need to know what things are being done in the antenatal visits and understand what their wives are undergoing.” – primigravida, age range 21–30 years.*

*“My husband and family members helped me get to the hospital and advised me on what diet to take during my pregnancy.” – primigravida, age range 31–40 years.*

*“In couple counselling, we educate the husband on what to expect during the antenatal visits and what do expect during delivery. We expect that the husband will tell their extended family about the hospital visits.” – HCW, age range 41–50 years.*

Working mothers, in general, reported that their colleagues were supportive despite not having official obligations to allow a mother to take leave from work to attend the ANC.

*“We don’t have specific entitlement to take leave to make these hospital visits but my boss has been supportive so far.” – primigravida, age range 31–40 years.*

*“I don’t take leave from the boss. I adjust my work among co-workers. All of them are male co-workers… but so far they have never said no when I had to excuse myself from work to come for the hospital visit.” – primigravida, age range 21–30 years.*

To reach the ANC services to women residing in rural areas and villages, the health workers reported the need to work in collaboration with local leaders.

*“I think it is a good idea if the Gup (village headman) invite us to give health talks in their village meetings.” – HCW, age range 41–50 years.*

### Perceived barriers for early ANC booking:

- Geographical inaccessibility

In rural areas, physical connectivity influenced the access to ANC services, as one HCW described a situation in Gasa district:

*“The footpath between the health centre and the village would remain under snow for several months in winter during which we did not offer any ANC services or attend to deliveries. Now, the Village Health Workers are connected to the Health Assistants through mobile phone.” – HCW who served in Lunana.*

*“In the rural areas where women have to walk long distances to the health centres, they would come only for few visits.” – HCW who served in rural areas for 13 years.*

b) Gender sensitivity

Gender sensitivity was recognised as a barrier by some healthcare workers, as shown below:

*“Coming to hospital and to get checked by male health workers was a major challenge.” – HCW who served in Lunana.*

*“Some women are shy and cannot face the healthcare workers and talk about their pregnancy.” – HCW, age range 41–50 years.*

c) Cost/time investment

The cost for having to make multiple visits to health centres was a barrier where one mother deliberately delayed her booking visit:

*“If I come for the first visit at three months, then the nurses will tell me that I have to come monthly thereafter. So I have to make more visits. To make visits, there are problems of lack of time and sometimes money. My friends also had lack of time so they also came around five or six months only. I cannot afford to come to the hospital time and again when there is nothing wrong with the baby and myself.” –second gravida, age range 21–30 years.*

d) Documentation for registration

In the cases of single motherhood or unstable relationships, the inability to produce the documents of the husband during the registration for ANC was seen as a factor for late booking.

*“There are some who do not make it to early antenatal check-ups. These are mostly those who are unmarried, or those who do not have a stable relationship with their partner. They would be involved in social issues with their parents or the partner’s parents.” – HCW, age range 31–40 years.*

However, to allow access to ANC, the documents of the husband is not mandatory though the ANC booklet is linked to the civil registration system in the country:

*“There are who come late for booking. They are usually students or those who are not legally married. However, if she is unable to produce the documents of the father, we don’t force it either.” – HCW, age range 31–40 years.*

### Contradictory information about the time of booking

While the healthcare workers gave information on the need to have early booking in the subsequent pregnancy:

*“We provide counselling to the mothers in the current pregnancy to encourage early booking in the next.” – HCW, age range 41–50 years.*

But the mothers claimed that no such information was given:

*“They told me that I should come to hospital [in the next pregnancy]. But nothing about the timing of the first visit.” – fifth gravida, age range 31–40 years.*

*“In my last pregnancy, the health workers told me to use contraceptives and nothing about when to come to hospital [for booking ANC].” – second gravida, age range 21–30 years.*

Additionally, while healthcare workers expressed the notion that multigravidae women would come for early ANC in the next pregnancy:

*“For the primi-mothers, it is their first time and they don’t even understand what’s happening to them. They don’t understand the importance of ANC and what things are done in the ANC. (…) The multi-mother, with their past experience of having given birth before, they come to us as soon as they have confirmed their pregnancy.” – HCW, age range 41–50 years.*

mothers did not necessarily echo this sentiment, as described in the words of one mother:

*“What you read and learn about pregnancy and what you experience in pregnancy is very different. With all these experiences, I may need less support in my next pregnancy.”* – *primigravida, age range 31–40 years.*

While healthcare workers promoted early booking because of its importance in maternal and child healthcare, one of the underlying drivers of early booking was also the need to achieve at least eight antenatal visits during the pregnancy period.

*“If we don’t book [the pregnant mothers] early, we cannot achieve eight visits and then our centre will be reflected amongst the poor performers.” – HCW, age range 41–50 years.*

## Discussion

While Bhutan has focused on achieving the target of providing at least eight ANC visits over the course of a pregnancy since 2010, this is the first study in the country to describe the magnitude and determinants of women not accessing ‘early’ ANC. Two women out of every three receiving care for a delivery or miscarriage had either a late or no antenatal booking, while almost everyone was covered with at least one ANC visit during the pregnancy. Women with only primary education and those residing in rural areas were more likely to present late for the first ANC contact. While both healthcare workers and mothers recognized the importance of (early) ANC coverage, the in-depth interviews identified several context-specific barriers, including geographic, socio-cultural and administrative aspects.

The coverage of early ANC in this study was similar to that of 24% in LMICs [2, 21] and much lower than that of 50% reported in Nepal in 2011 [3]. Though the health network in Bhutan has a coverage where more than 95% of the total population live within 3 hours walking distance from a health facility [22], women in rural areas were less likely to receive early ANC [3, 21, 23, 24]. Physical inaccessibility was reported to be an important factor given the Himalayan terrain and the limited coverage of the road networks [3, 21, 23, 24]. In such situations, the health workers reported that primary health care ORCs were highly effective, though challenging, in taking the services to the scattered population as reported in Nepal [3].

The other important determinant of early booking was the level of education, as also evidenced in other studies [2, 3, 21, 25], which in many cases was linked with higher socioeconomic status and empowerment of women for family planning and pregnancy choices [26]. Given the direct and indirect costs of ANC visits, women with higher education had higher acceptance of the need for early ANC and the preventive aspect of maternal and foetal problems [26]. This likely played a larger role than the direct effect of education, as most women expressed that school education did not contribute to health information about early ANC coverage.

Women visiting the health centres for ANC received health education about the importance of early ANC in the next pregnancy. Healthcare workers expressed the expectation that women who have given birth before would present early for booking in the next pregnancy as reported in other studies [24, 25]. However, the quantitative findings and qualitative findings among pregnant women contradicted this notion and suggested that multigravida women are more likely to have a late booking, possibly because they deemed themselves more experienced and less in need of support; similar findings were reported elsewhere [27]. This is noteworthy, as in general, women suggested that their peers (friends, family) were among the main sources of information, and played a key role in the decisional process of when and where to seek ANC. This may have resulted in the observed mixed and sometimes incorrect messages about the timing of the first ANC visit, similar to that reported from interviews [27], from friends and relatives who had delivered before.

While there has been considerable advocacy on the prevention of teenage pregnancy, the ANC package does not address the specific needs of teenage mothers in Bhutan. For teenage mothers and others who cannot produce the documents for the father of the child, the misconception that these documents are required caused further delays in seeking ANC. On a similar note, Adolescent Friendly Health Services for general and antenatal care are available only at the National Referral Hospital and selected district hospitals.

One of the reasons reported at the provider level for early ANC booking was with an aim to achieve at least eight ANC visits, which is a performance indicator in the national MCH Programme. However, the number of visits does not reflect early booking in all cases. There are situations where eight ANC visits are documented but with the booking visit in the third trimester, completely missing out on the importance of early ANC booking while still achieving the nominal targets.

The health workers also reported on the unmet need for female health workers in the delivery of ANC services. Shyness to be examined by a male health worker was an important factor in delayed first ANC contact, especially among teenage and primigravida mothers. While many other countries have implemented the usage of female health workers in the primary health care MCH activities [3, 28], the Ministry of Health has been unable to deliver this despite public and community demands.

From our findings, considering the key role that experienced peers play in the information and decision-making process, we recommend the MCH Programme to develop a comprehensive information package for aspiring parents. A focus on how women with experience of pregnancy communicate key messages with other pregnant women in their personal environment would be of great value in such a package. Additionally, there is a need to deploy female Health Assistants and nurses in the primary level MCH activities. Health education also needs to target the family members who are decision-makers in the family [26], ideally including through the use of the popular national television and social media.

The findings are a triangulation of quantitative observations of the study, reported using the Strengthening the Reporting of Observational Studies in Epidemiology (STROBE) guidelines [29], and qualitative findings reported using the ‘Consolidated Criteria for Reporting Qualitative Research’ (COREQ) guidelines [30]. One limitation of our study is that the study population consisted of women delivering at the hospital, which may be a population with a higher rate of complications than women delivering at the primary healthcare level (Basic Health Unit) or delivering at home, and may also be a population that is more likely to attend ANC. We anticipate however that this bias will be limited, as the hospital-based deliveries represent 58% of all deliveries in Bhutan and nearly half of all women who delivered in these three hospitals had their ANC at primary healthcare centres [13, 18]. Additionally, our sample for qualitative interviews did not include those women who did not have an ANC booking, and thus could not explore their reasons for having no ANC. Another limitation of this study is that the qualitative interviews with the pregnant mothers were conducted by a male doctor, which may have contributed to social and gender sensitivity bias. To offset for the asymmetry of power between the interviewer (doctor) and the mother, the formal interview, however, started only after the mothers sufficiently understood that the interviewer was not their treating doctor. The interviewer did not wear a white coat and stethoscope at the time of the interview. The interviews were conducted in local language and then transcribed into English; some degree of interpretation of information could have happened at the stage of transcription. The interviews of two participants were done in the presence of companions (husband, sister), which could also have limited the free expression of their opinions. Lastly, the interviews were conducted in a period of 3 months (May–August) and could not capture the time period of seasonal variation, where the roads are disrupted by monsoon rains in the summer and blocked by ice and snow in the winter, which influences the accessibility of health services.

## Conclusion

The magnitude of late antenatal booking in Bhutan was high and appeared to be associated with educational, geographic, socio-cultural and administrative characteristics, at the levels of the individual expecting mothers, the health system, and the general context. The barriers to early ANC are both a challenge and an opportunity to review and strengthen the maternal and child healthcare delivery policies and programmes. From the monitoring perspective, there is a need to assess the ‘early’ ANC coverage rather than counting only the number of ANC visits.

## Additional files

Additional file 1: Data extraction pro forma ANC booking study Bhutan. (DOCX 23 kb)

Additional file 2: Interview guide for in-depth interviews on the themes around late antenatal booking (DOCX 15 kb)

## Abbreviations

ANC: Antenatal care
HCW: Healthcare worker
LMIC: Low- and middle-income countries
MCH: Maternal and child health
ORC: Out-reach clinics
PR: Prevalence ratio
WHO: World Health Organization
