# Supplementary material for: “If we miss this chance, it’s futile later on” – late antenatal booking and its determinants in Bhutan: a mixed-methods study
Source: BMC Pregnancy Childbirth. 2019 May 7;19:158. doi: 10.1186/s12884-019-2308-5 (PMC6505275; doi:10.1186/s12884-019-2308-5)
Supplement: Supplementary file 1 — Data extraction pro forma ANC booking study Bhutan. (DOCX 23 kb) [file 12884_2019_2308_MOESM1_ESM.docx]

**Supplementary file 1**

**Data extraction pro forma**

**The magnitude and determinants of late antenatal booking in Bhutan**

- For women who *deliver* or are *treated for pregnancy complications leading to termination of pregnancy (miscarriages)* at the three tertiary hospitals during 15 May to 14 August 2018. **PLEASE INCLUDE ALL WOMEN**.
- For any queries during filling up of this form, please call Dr Thinley Dorji.
- **ALL INFORMATION TO BE EXTRACTED FROM THE MCH HANDBOOK OR THE MCH REGISTER**

| **Sl no** | **Variable** |  |
| --- | --- | --- |
|  | HOSPITAL CODE | ⬜ Maternity Ward, JDWNRH  ⬜ GJP Birthing Centre, JDWNRH  ⬜ Maternity Ward, CRRH  ⬜ Maternity Ward, ERRH |
| 1 | MCH Registration number |  |
| 2 | Hospital admission number |  |
| 3 | Date of booking at the ANC unit |  |
| 4 | Date of birth of mother |  |
| 5 | Level of education of mother | ⬜ None  ⬜ Non-formal education  ⬜ Primary education  ⬜ Secondary education  ⬜ Graduate education  ⬜ Others |
| 6 | Residence | ⬜ Urban  ⬜ Rural |

|  | **Past medical history** |  |
| --- | --- | --- |
| 7 | Pre-existing hypertension | ⬜ No  ⬜ Yes |
|  | Known substance abuse (including heavy alcohol drinking) | ⬜ No  ⬜ Yes |
|  | Diabetes | ⬜ No  ⬜ Yes |
|  | Tuberculosis | ⬜ No  ⬜ Yes |
|  | Hepatitis B sAg | ⬜ Non-reactive  ⬜ Reactive |
|  | HIV status | ⬜ Non-reactive  ⬜ Reactive |
|  | Past obstetric history |  |
| 8 | Mother is Rh negative and father is Rh positive | ⬜ No  ⬜ Yes |
|  | History of three or more consecutive spontaneous abortions | ⬜ No  ⬜ Yes |
|  | Baby weighing less than 2500 grams | ⬜ No  ⬜ Yes |
|  | Baby weighing more than 4500 grams | ⬜ No  ⬜ Yes |
|  | Last pregnancy: admission for hypertension, preeclampsia, eclampsia | ⬜ No  ⬜ Yes |

|  | Previous surgery on reproductive tract (caesarean section, cervical cerclage, cone biopsy, myomectomy, ectopic pregnancy) | ⬜ No  ⬜ Yes |
| --- | --- | --- |
|  | Pregnancy description |  |
| 9 | Gravida |  |
|  | Parity |  |
|  | Living children |  |
|  | Abortion in previous pregnancy |  |
|  | Stillbirth in previous pregnancy |  |
|  | Death of any previous child |  |
| 10 | Number of ANC visits |  |
| 11 | Date of delivery or miscarriage |  |
| 12 | Gestational age at delivery |  |

-End-
