# Supplementary material for: “If we miss this chance, it’s futile later on” – late antenatal booking and its determinants in Bhutan: a mixed-methods study
Source: BMC Pregnancy Childbirth. 2019 May 7;19:158. doi: 10.1186/s12884-019-2308-5 (PMC6505275; doi:10.1186/s12884-019-2308-5)
Supplement: Supplementary file 2 — Interview guide for in-depth interviews on the themes around late antenatal booking (DOCX 15 kb) [file 12884_2019_2308_MOESM2_ESM.docx]

**Supplementary file 2**

**Interview guide for in-depth interviews on the themes around late antenatal booking**

**The magnitude and determinants of late antenatal booking in Bhutan**

**For *pregnant women***

1. Describe your experience with antenatal care services so far?
2. What is your personal opinion about attending antenatal care clinics during pregnancy?
3. When was your first antenatal care visit? Who was involved in this decision?
4. Does the Maternal and Child Health Programme or the hospital advise you on the timing of booking of pregnancy? From where have you heard about antenatal care services?
5. What do you know about coming early and late for antenatal care services during pregnancy?
6. What are the areas of support that are required to enable early visit to hospital for antenatal care pregnancy (husband, family, co-workers, healthcare providers)?

**For *ANC providers***

1. Describe your experience in providing antenatal care services so far?
2. What is your personal opinion on the importance for women to attend antenatal care clinics during pregnancy?
3. When do you think is the appropriate timing of the first visit to the antenatal care?
4. What do you think about the timing of the first antenatal care visit of your clients? Who do you think are involved in this decision?
5. What does the Maternal and Child Health Programme or the hospital advise women on the timing of booking of pregnancy? How?
6. What do you know about ‘early and late booking’ of pregnancy?
7. What are the areas of support that are required to enable early booking of pregnancy?
